# Strong Ferromagnetically-Coupled Spin Valve Sensor Devices for Droplet Magnetofluidics

**DOI:** 10.3390/s150612526

**Published:** 2015-05-27

**Authors:** Gungun Lin, Denys Makarov, Oliver G. Schmidt

**Affiliations:** 1 Institute for Integrative Nanosciences, IFW Dresden, Helmholtzstr. 20, 01069 Dresden, Germany; E-Mails: d.makarov@ifw-dresden.de (D.M.); o.schmidt@ifw-dresden.de (O.G.S.); 2 Material Systems for Nanoelectronics, Chemnitz University of Technology, Reichenhainerstr. 70, 09107 Chemnitz, Germany

**Keywords:** droplet microfluidics, spin valve, ferromagnetic coupling, high field sensing, ferrofluid

## Abstract

We report a magnetofluidic device with integrated strong ferromagnetically-coupled and hysteresis-free spin valve sensors for dynamic monitoring of ferrofluid droplets in microfluidics. The strong ferromagnetic coupling between the free layer and the pinned layer of spin valve sensors is achieved by reducing the spacer thickness, while the hysteresis of the free layer is eliminated by the interplay between shape anisotropy and the strength of coupling. The increased ferromagnetic coupling field up to the remarkable 70 Oe, which is five-times larger than conventional solutions, brings key advantages for dynamic sensing, e.g., a larger biasing field giving rise to larger detection signals, facilitating the operation of devices without saturation of the sensors. Studies on the fundamental effects of an external magnetic field on the evolution of the shape of droplets, as enabled by the non-visual monitoring capability of the device, provides crucial information for future development of a magnetofluidic device for multiplexed assays.

## 1. Introduction

Modern medical research calls for the development of new technologies or platforms. Droplet microfluidics is a prominent example of such new platforms and has picked up momentum in recent years [1]. Droplet microfluidics bears great potential for the practical realization and replacement of conventional medical instrumentations for, e.g., protein [2] and DNA assays [3], drug discovery [4] and cell studies [5]. Concurrently, the recent advances in droplet micro-magnetofluidics have witnessed growing interest in the utilization of magnetic materials [6,7]. For instance, droplets encapsulating ferrofluids allow on-chip manipulation of biochemical species [8] and remote actuation of liquid by coupling with an external magnetic field [9,10]. Liposome capsules or vesicles associated with magnetic particles have been broadly studied for targeted drug delivery [11,12]. Furthermore, magnetic particles have been utilized as droplet barcodes, either providing an additional functionality of manipulation [13,14] or enhancing the encoding capacity [15] for multiplexed assays. Hence, developing on-chip magnetic field sensing technologies in droplet microfluidic systems to actively sense and analyze droplets containing various amounts of magnetic materials is highly demanded and desirable.

Magnetoresistive sensing technologies have been adopted in biosensing applications since the end of last century [16]. Major advantages of the employment of magnetoresistive sensorics are associated with compact integration, direct signal readouts, the capability to detect magnetic labels, as well as providing a feedback control for on-chip actuation with magnetic materials. On-chip magnetoresistive sensing reveals great potential in microarray technologies [17,18], which relies on static detection of magnetic labels bound to the substrate’s surface for molecular recognition [19]. Recently, there has been a trend towards in-flow detection of magnetically-functionalized objects in microfluidics [20], which is superior in terms of throughput compared with the conventional static detection schemes [21] when objects of interest are immobilized at the sensor’s location for the detection event. While sensing of magnetic particles, beads or cells in the flow is rather advanced already [22], the development of magnetoresistive sensing technologies for droplet microfluidic systems is still in its infancy. To date, droplet-based micro-magnetofluidic systems have employed spin valve sensors [23] and giant magnetoresistive (GMR) multilayers [15,24,25] to determine the size and magnetic content of droplets.

Spin valve sensors are an advanced type of GMR sensorics due to its large sensitivity to small magnetic fields. However, spin valve sensors are commonly applied as switches, and they are characterized with a hysteresis that is not suitable for dynamic sensing in fluidics due to its vulnerability to random magnetic fields and magnetic noise [26]. Typical approaches to eliminate the hysteretic response of spin valves involve biasing a free layer’s magnetization with shape anisotropy, which is orthogonal to a pinned layer’s magnetization set by exchange bias [27] or designing a sensor with a yoke shape geometry to eliminate domain walls [26]. Alternatively, an external magnetic field is used to stabilize the free layer’s magnetization during the measurements [28]. The free layer and the pinned layer are usually weakly coupled, so as to bring the sensing point to a near zero magnetic field [29]. In this respect, in order to detect superparamagnetic nanoparticles or beads, which are frequently used as the carrier materials for labeling or manipulation of species in fluidics, an external out-of-plane magnetic field is applied to induce net magnetic moments. Nonetheless, particularly for applications, such as to measure the size of ferrofluid droplets by a magnetofluidic device [23,24], an in-plane-dominant magnetic field is typically employed. In such cases, a stronger in-plane biasing field is crucial to achieve larger detection signals. The low field sensitive feature of spin valve sensors is vulnerable to saturation during the field biasing procedure and limits the further increase of detection signals. Therefore, a magnetic field sensor with a larger biasing point is advantageous and facilities the operation of the device in practical settings.

In this article, we report a magnetofluidic platform for in-flow detection of ferrofluid droplets in microfluidics. For this purpose, we optimize the performance of the integrated spin valve sensor device to be hysteresis free and to possess a biasing point shifted to a remarkable 70 Oe, which is at least five-times larger than applied previously [23]. A strong ferromagnetic coupling between the free layer and the pinned layer of spin valve sensors is achieved by reducing the spacer thickness between pinned and free layers of the spin valve stack, while a reduced hysteresis is achieved by tailoring the shape anisotropy and the coupling strength that is crucial for on-chip dynamic sensing. The optimized spin valve sensors integrated in a microfluidic device allow the detection of ferrofluid droplets with about six-times enhanced signal responses compared to the commonly-used GMR multilayers with the same measurement configuration [24]. In addition, the magnetofluidic device provides insights into the fundamental effects of an external magnetic field impact on the shape evolution of droplets in microfluidics. In turn, the fundamental understanding provides crucial information for the future design of micro-magnetofluidic devices for multiplexed magnetic barcoding assays.

## 2. Experimental Section 

### 2.1. Fabrication of Spin Valve Sensors

The spin valve sensors are prepared by magnetron sputter deposition on thermally-oxidized silicon wafers. The fabrication process can be described as follows: Firstly, the substrate was cleaned with acetone and isopropanol, rinsed by DI water and placed on a hotplate at 120 °C to get rid of the absorbed vapor. Then, the surface of the substrate was patterned with photoresist by photolithography. For the lithography process, a photoresist (AZ5214E, Microchems) was coated on the substrate. The spin speed and time are 4500 rpm and 35 s. After the coating, the substrate was transferred on to a hotplate and baked at 90 °C for 4 min to remove residual solvents. Then, the substrate was transferred to a mask aligner (Karl Suss, MJB4) and exposed to UV light for 2 s. A photomask with designed chromium patterns of sensor geometries was used. Subsequently, the wafer was placed on a hotplate again, which was set at 120 °C for 2 min. Afterwards, it was exposed at the mask aligner without a photomask for 30 s and developed by a developer (MIF 726, Microchems) for 1 min to remove the uncrosslinked photoresist. 

The substrate with patterned photoresist was introduced into the chamber with a base pressure 9.5 × 10^−8^ mbar, where the deposition of the GMR layer stack was carried out. The layer stack used in this study is: SiO_x_/Ta (5 nm)/Py (4 nm)/CoFe (1 nm)/Cu (*t* nm)/CoFe (1 nm)/Py (4 nm)/IrMn (8 nm)/Ta (2 nm), with *t* the thickness of the Cu spacer and Py = Ni_81_Fe_19_. Argon was used as the sputter gas, with a sputter pressure of 9.5 × 10^−4^ mbar and a flow rate of 10 sccm kept constant during the deposition. To induce exchange bias in spin valve sensors, an external in-plane magnetic field of about 0.5 kOe was applied during the deposition by means of arrangements of permanent magnets behind the sample holder. The thickness of each deposited layer of spin valve sensors is controlled by adjusting the deposition time with pre-calibrated deposition rates. After the deposition of the sensor stack, a lift-off process was used to remove the photoresist. Then, another photolithography is performed to align the electrical contacts with the sensors. In this case, Ta (5 nm)/Cu (200 nm)/Ta (5 nm) were deposited as the electrical interconnects by magnetron sputtering.

### 2.2. Assembly of Microfluidic Devices

The microfluidic channel was fabricated by casting PDMS (SYLGARD 184, 1:10 wt% for curing agent and base polymers) with a SU-8 50 (Microchem) mold. The mold was prepared by coating the photoresist SU-8 50 on a silicon wafer at a spin speed of 1000 rpm for 35 s leading to the height of the channel of 100 µm. The photoresist SU-8 50 was cured at 60 °C for 5 min and at 90 °C for 15 min. Then, the wafer with cured SU-8 50 was illuminated by UV light with a mask aligner (MJB4, Karl Suss) and baked at 90 ° C for 15 min. The channel geometry was revealed by immersing the wafer in a solvent (mr-Dev 600, micro resist technology GmbH) to remove unexposed areas. The wafer was cleaned by isopropanol and dried by compressed air.

Before the assembly of the channel and the sensor chip, a layer of polyetherimide (PEI) of a thickness of about 300 nm was spin coated on top of the sensors as an insulation layers. Afterwards, a layer of SiO_2_ of 200 nm was deposited on top of the PEI by e-beam evaporation in order to facilitate bonding with the PDMS channel. To achieve permanent bonding, the PDMS microfluidic channel and the sensor chip were activated in an oxygen plasma (40 mW, O_2_ flow rate: 20 sccm) for 30 s. They were brought in touch and aligned with each other under a microscope.

### 2.3. On-Chip Formation of Ferrofluid Droplets

The microfluidic channel is composed of a T-junction to produce droplets. Ferrofluid magnetic nanoparticles (EMG 700 series, purchased from Ferrotec) are used as a disperse phase, and mineral oil (Sigma-Aldrich, M8410) with 5% surfactant (SPAN 80) are used as a continuous phase. The ferrofluids are dispersions of superparamagnetic nanoparticles with a nominal size of 10 nm. Different concentrations (5.0 mg/mL and 7.5 mg/mL) of ferrofluids were prepared by diluting the original stock solutions with DI water. The oil and ferrofluid particles are separately loaded in two syringes and pumped into the microfluidic channel by a syringe pump (Cetoni GmbH, NEMESYS). Water-in-oil emulsion droplets encapsulating ferrofluid particles are thus produced at a T-junction directly on-chip. For the present study, the flow rates of oil and ferrofluids were kept in total at 15 nL/s with a flow speed of droplets about 1 mm/s. To produce droplets of different sizes (volume: from 200 pL to a few nanoliters), the ratio of the flow rates of the two different fluids was adjusted accordingly.

### 2.4. Magnetoelectrical Characterizations of Spin Valve Sensors

For magnetoelectrical characterizations, a sample holder with four pins was used to measure the resistance of sensors by the standard four-point method. The sensor was clamped in the holder, which was placed in between the pole-shoes of an electromagnet. An external uniform magnetic field was applied on the sample, and the sample resistance was recorded for each magnetic field by a multimeter (Keithley Model 2000). For the measurement, the magnetic field was cycled between ±300 Oe to measure the full range GMR curves.

### 2.5. Real-Time Electrical Measurements of Droplets in Microfluidics

An electrical measurement setup was built up to detect droplets in the microfluidic channel. A lock-in amplifier (SRS 830) was used to produce AC measuring current of about 1 mA to a Wheatstone bridge circuit. The bridge circuit was formed by the spin valve sensor for the detection of droplets together with other resistors. The Wheatstone bridge allows us to reduce the background signal level, so that the sensitivity can be enhanced. For signal amplification, the differential voltage of the bridge was picked up by the lock-in amplifier. In this case, a reference sinusoidal signal was used to modulate the AC voltage signal at a high frequency up to 1 kHz to increase the signal-to-noise ratio. After signal processing by the lock-in amplifier, the analog output voltage was fed into a data acquisition box (NI-USB 6009) with a sampling rate of 5 kHz. As the ferrofluid is made of superparamagnetic particles, an external magnetic field was applied to magnetize the particles by using a permanent magnet placed below the sensor. The position of the magnet was carefully adjusted to bias the sensor to the most sensitive region via monitoring the real-time detection signals of droplets by a LabVIEW program.

## 3. Results and Discussion

### 3.1. Optimization of Spin Valve Sensors

**Figure 1:**
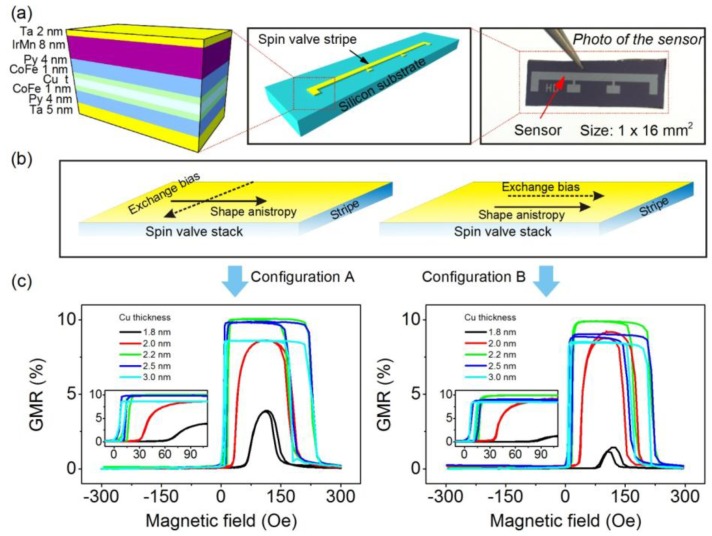
(**a**) Schematic representation of the layer stack of spin valve sensors (left) and a sensor stripe on a silicon substrate (middle), as well as the photograph of a spin valve sensor patterned with a size of 1 × 16 mm^2^ (right); (**b**) Schematics of two anisotropy configurations of spin valve sensors, which are achieved by setting the direction of exchange bias with respect to shape anisotropy. For Configuration A (crossed anisotropy), the exchange bias is along the short axis of the sensor stripe, and hence, it is perpendicular to the direction of the shape anisotropy. For Configuration B (parallel anisotropy), it is along the long axis of the sensor stripe parallel to the direction of the shape anisotropy; (**c**) Giant magnetoresistive (GMR) curves for the spin valve sensors prepared with crossed and parallel anisotropy configurations. The spin valve sensors are of the same layer stack structure as shown in (a) and the thickness of Cu ranges from 1.8 nm to 3.0 nm. Inset: The GMR response of the free layer. The x- and y-axis correspond to those in the main figure.

To achieve a hysteresis-free sensor response, the magnetization of the free layer should reverse via a coherent rotation process [27]. The strength of interlayer exchange coupling, as well as the anisotropy configuration play a crucial role in the magnetization reversal mode of spin valve sensors [30]. The former is determined by the thickness of the spacer layer of spin valve sensors [31,32]. To optimize the coupling strength aiming at the increase of the biasing point of the sensor as needed for dynamic detection, we prepared a series of spin valve sensors with different thicknesses of the Cu spacer in the range from 1.8 nm to 3.0 nm (Figure 1a). For the experiments on the sensor optimization, the layer stack was patterned into rectangular stripes with a size of 1 × 16 mm². The exchange bias direction of the sensors was set during the deposition, which is either along the long axis or the short axis of the sensor stripes (Figure 1b). The interlayer exchange coupling between the free layer and the pinned layer thus creates an induced uniaxial anisotropy, which is parallel to the pinning direction (the exchange bias direction). On the other hand, the large aspect ratio of the sensor stripe induces a shape anisotropy along the long axis of the sensor stripe. Thus, according to the arrangements of the exchange bias direction with respect to the shape anisotropy, two configurations of spin valve sensors with crossed or parallel anisotropies can be realized.

**Figure 2:**
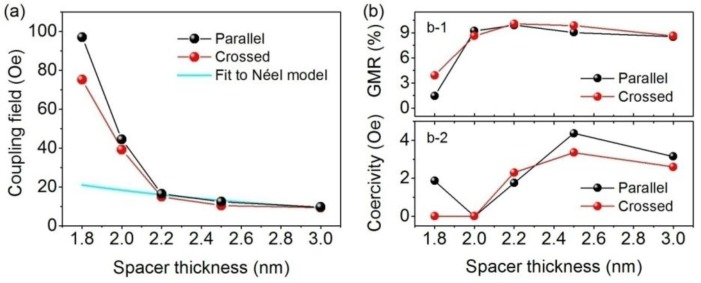
(**a**) Ferromagnetic coupling field as a function of the spacer thickness for spin valve sensors with the parallel anisotropy configuration, as well as the fit of the data to the Néel model. Lines connecting the symbols are a guide to the eyes; (**b**) GMR ratio (b-1), as well as the coercivity of the free layer (b-2) as a function of the spacer thickness for spin valve sensors of parallel and crossed anisotropy configurations.

The GMR curves of spin valve sensors with the two different anisotropy configurations are shown in Figure 1c. With the reduction of the thickness of Cu from 3.0 nm to 1.8 nm, the exchange coupling strength, as characterized by the shift of the free layer’s response curves, of both series of spin valve sensors increases (Figure 2a). As the shift of the free layer’s response curves is opposite the pinning direction (in the negative direction of the magnetic field), the exchange coupling is ferromagnetic. It was observed that the ferromagnetic coupling fields for the sensors pinned along the short axis of the stripes (crossed anisotropy configuration) are smaller than those pinned along the long axis of the stripes (parallel configuration), which can be attributed to magnetic stringing fields emanating from the pinned layer. 

The ferromagnetic coupling field typically arises from two contributions: orange peel coupling and direct coupling through pinholes. The orange peel coupling can be described in the frame of the Néel model [33]:

(1) $$H_{coupling}=M\frac{\pi^{2}}{\sqrt{2}}\frac{\gamma^{2}}{\lambda }\frac{1}{t_{F}}\exp(\frac{-2\pi \sqrt{2}t_{s}}{\lambda })$$

with M being the magnetization of the pinned layer, t_F_ and t_s_ being the thickness of the free layer and the spacer layer, λ and γ being the wavelength and the magnitude of correlated roughness, respectively. The Néel model can be successfully applied to fit to the experimental data with spacer thickness ≥2.2 nm (Figure 2a) for sensors designed with a parallel anisotropy configuration (without stringing field coupling). A wavelength λ ~ 13 nm and a roughness γ ~ 0.23 nm are determined, which are close to the same parameters extracted from similar spin valve sensors [34]. For Cu thicknesses smaller than 2.2 nm, a large deviation from the Néel fit is observed. Hence, the strong coupling field could be ascribed to the formation of pinholes in the Cu spacer [34]. 

With the increased ferromagnetic coupling strength, a hysteresis-free sensor response of the free layer is achieved (Figure 2b). Although characterized by a smaller GMR ratio (Figure 2b-1) compared with other hysteresis-free sensors of larger spacer thickness (2 nm), the spin valve sensor with the Cu thickness 1.8 nm and crossed anisotropy shows the largest coupling field (about 70 Oe) and a rather broad sensing range. These features are advantageous for magnetic in-flow detection, *i.e*., a large external magnetic field can be applied to magnetize the particles to induce a larger magnetic moment and, thus, to enhance the detected signals without saturating the sensor.

### 3.2. Dynamic Monitoring of Droplets in Microfluidics by Spin Valve Sensors

**Figure 3:**
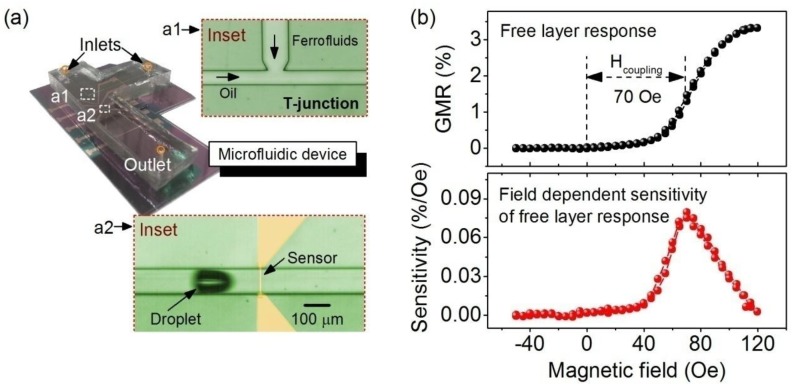
(**a**) Photograph of an assembled microfluidic device with an integrated on-chip T-junction (a1) and spin valve sensors (a2). (a1) and (a2) are micrographs corresponding to the dashed squares indicated on the photograph of the device; (**b**) GMR response of the free layer of an integrated spin valve sensor with a spacer thickness of 1.8 nm with a crossed anisotropy configuration (top). The sensitivity is plotted as a function of the magnetic field (bottom).

For magnetic detection, the optimized spin valve sensor of a crossed anisotropy configuration and Cu thickness of 1.8 nm was integrated in a microfluidic channel (Figure 3a). It has been demonstrated previously [24,35] that the real-time detection of the peak patterns of droplets loaded with ferrofluids using a GMR sensor carries the signature of the emulsion droplets. The signature can be resolved in terms of the signal amplitude and peak width to analyze encapsulated magnetic content, as well as droplet size, respectively. A magnetoresistive emulsion analyzer [35] with the size of the GMR sensor along the fluid flow direction being 1 mm is capable of analyzing bivariant droplets, but with the size limited to larger than 1 mm (volume: about 110 nL). This limitation is due to the fact that the peak width is no longer sensitive to the change of droplet size when the droplet is smaller than the sensor size. To address the need to analyze bivariant nanoliter or even picoliter-droplets in microfluidics, we fabricated spin valve sensors with a size of 6 × 100 µm^2^ (6 µm along the fluid flow direction), which is smaller than the length of droplets typically produced in microfluidics. The aspect ratio of the integrated spin valve sensors is close to the above sensors used for optimization; hence, similar performance is expected. The transfer curve of the integrated spin valve sensor (Figure 3b) shows that the sensor retains a GMR ratio of about 3.5% close to the optimized spin valve stack of the same spacer thickness and possesses a maximum sensitivity of about 0.08%/Oe at a large field of about 70 Oe. The sensitivity of the sensor is comparable to previously reported spin valve sensors (with a sensitivity of 0.077%/Oe) employed for the detection of ferrofluid droplets [23]. However, the ferromagnetic coupling field is about five-times larger than the reported value of about 15 Oe [23]. 

**Figure 4:**
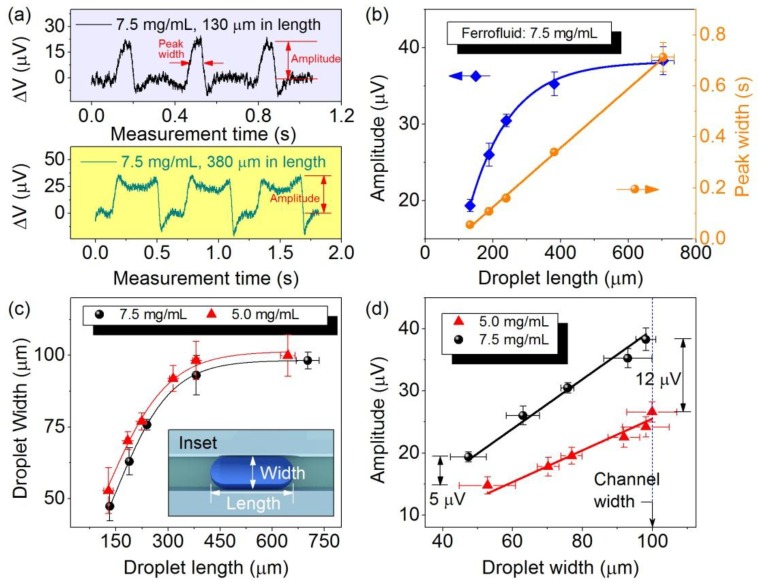
(**a**) Real-time detection of emulsion droplets with different lengths (130 µm and 380 µm), but the same concentration (7.5 mg/mL) of loaded ferrofluids; (**b**) The detection signal amplitude (square) and peak width (sphere) as a function of droplet length (with volume ranging from 200 nL to a few nanoliters). Lines are a guide to the eye; (**c**) The droplet width as a function of the droplet length for different concentrations of encapsulated ferrofluids. Lines are a guide to the eye. The inset depicts the schematics to measure the width and length of droplets; (**d**) The signal amplitude as a function of the droplet width for different concentrations of encapsulated ferrofluids. Lines are linear fittings to the data. Error bars are standard deviations of measured data.

Figure 4a shows the real-time detection results of ferrofluid emulsion droplets produced with different lengths. Each peak represents the detection event of an emulsion droplet. Only local stray fields are detected by the sensor. The local maximum and minimum of the detection peaks are attributed to the local maximum and minimum of the stray field distribution around the sensor. The amplitude of the detection signals, e.g., while measuring droplets of a volume ~1 nL containing ferrofluids of 7.5 mg/mL is about six-times that of a previously reported microfluidic device (about 5 µV) with integrated GMR multilayers in detecting the same amount of ferrofluids based on the same measurement configuration, which possesses a maximum sensitivity of 0.4%/Oe at a low biasing field of 12 Oe [24].

The temporal interval between the rising and falling edges of detection signals is referred to as the detection peak width. The values of the peak width can be robustly extracted by a peak search algorithm without artifacts [36]. Figure 4b shows that the length of droplets (with a volume ranging from 200 pL to a few nanoliters) is proportional to the detection peak width, which is consistent with previously reported results [24]. In contrast to the expectation, the signal amplitude changes with the droplet length, namely we observe that it increases with the increase of the droplet length with a clear trend to saturate at the higher range. The droplet length of >100 µm along the travel direction is larger than the sensor size (6 µm). Hence, the change of the signal amplitude with the droplet length is ascribed to the stretching of droplets along the flow direction by an external magnetic force. This leads to the shrink of droplets in the direction transverse to the droplet travel. This statement is confirmed by microscopy. The external magnetic force results from an external magnetic field used to magnetize the superparamagnetic nanoparticles (Figure 4a,b). The effect of droplet deformation under an external magnetic field has been studied previously on sessile microliter-droplets [37]. It was shown that the deformation is determined by the balance of magnetic surface force, gravitational force and surface tension. Surface tension is an intrinsic property of a liquid’s interface with another contacting medium, which is referred to as the interfacial force per length or interfacial energy per area (density). In microfluidic measurements with nanoliter-droplets, magnetic force and interfacial energy are the main contributions to the final shape of droplets. With a constant external magnetic field of about 70 Oe, relatively large magnetic force is exerted on the droplets carrying larger concentrations of ferrofluids thus, giving rise to substantial shrinkage of the droplets (in-plane) perpendicular to the droplet travel direction. This is evidenced by the decrease of the droplet width when the concentration of ferrofluids is increased from 5.0 mg/mL to 7.5 mg/mL (Figure 4c). It is to be noted that the initial surface area of longer droplets is larger than the smaller ones. The interfacial energy to be overcome in order to stretch larger droplets to an equal extent is larger than smaller droplets. This mechanism has led to the effect of lateral shrinkage being almost extinguished for longer droplets (Figure 4c).

Figure 4d shows a linear relationship of the signal amplitude with the droplet width (Figure 4d). The ratio of the slope of the two curves (1.56 ± 0.05) obtained from droplets encapsulating ferrofluids of 5.0 mg/mL and 7.5 mg/mL can be determined by linear fits to the data. The value agrees well with the ratio of the different concentrations of ferrofluids encapsulated in the droplets (equal to 1.5). Considering the fact that magnetic stray fields decay quickly over distance and the distance from the bottom of the channel to the sensor (about 500 nm) is far smaller than the height of the channel (100 µm), only the magnetic stray fields generated from the lower part of the droplets (close to the location of the sensor) contribute mainly to the final detected signals. Thus, the detected signals are less dependent on the whole volume of droplets, but rather the surface coverage of magnetic nanoparticles, which is analogous to the linear dependence of detection signals on the surface coverage of magnetic particles by a GMR sensor in a static detection scheme [28]. In other words, the measured signal is determined by the area of the sensor exposed to the magnetic field stemming from the magnetized droplet. As droplets are close to the sensor, the area exposed to the field can be estimated by the area of the sensor covered by the droplet. Because the sensor along the flow direction is only 6 µm, it is completely covered by the droplets. Therefore, the surface coverage of particles is determined by the width of droplets, which is perpendicular to the flow direction. In agreement with this discussion and based on our experimental data (Figure 4d), the signal amplitude can be given by ΔV~ *c·w*, with *c* the concentration of ferrofluids and *w* the width of the droplet, namely the size perpendicular to the travel direction. 

The fundamental effect of the lateral shrinkage of droplets under an external applied magnetic field is crucial for magnetofluidic applications based on multiparametric analytics relying on magnetic droplet barcodes [36]. As the extent of shrinkage is a function of the droplet length and encapsulated concentration of ferrofluids, analysis based on two parameters, such as the amplitude and the peak width, to analyze the size and concentration of encapsulated magnetic content is still feasible, but needs a prior calibration with optical measurements of the size. The effect determines the minimum volume of droplets that can be still distinguished between droplet groups of two different concentrations. For instance, the difference in the signal amplitude between two groups of droplets carrying ferrofluids of 5.0 mg/mL and 7.5 mg/mL drops from 12 µV to 5 µV (Figure 4d), which may eventually cause the overlap of signal amplitudes when the size of droplets becomes even smaller (about 100 µm in length). Reducing the external magnetic field may alleviate this effect, but this will result in the reduction of the signal amplitude. However, our results suggest that the lateral shrinkage of droplets under an external magnetic field can be extinguished when droplets become longer (>500 µm) (Figure 4c) and only the lower part of droplets is contributing to the detected signals. Thus, the fundamental understanding of these influencing factors provides a solution for future development of a magnetofluidic platform to analyze and distinguish droplets of even smaller volumes, that is by integrating GMR sensors into a microfluidic channel with an even smaller cross-section to geometrically elongate droplets into longer plugs in order to avoid this issue.

## 4. Conclusions

We designed a microfluidic droplet analyzer based on a hysteresis-free spin valve sensor with a strong ferromagnetic coupling field. The coupling field of the spin valve sensor is 70 Oe, which is about five-times larger than that of conventional hysteresis-free spin valve sensors and GMR multilayers used for magnetic in-flow detection. The strongly ferromagnetically-coupled spin valve sensors with reduced hysteresis bring key advantages for magnetic in-flow detection: (i) a large in-plane/out-of-plane magnetic field can be applied to magnetize ferrofluid particles to induce large magnetic moments for sensing; (ii) it facilitates the operation of the device without easily saturating the sensor; and (iii) it allows improving the signal-to-noise ratio. We showed that with this device, the signal-to-noise ratio can be enhanced by six-times that of state-of-the-art realizations based on GMR multilayers. The device has been able to analyze bivariant droplets produced in microfluidics and to resolve the evolution of droplet size (volume: from ~200 pL to a few nanoliters) under an external applied magnetic field of up to 70 Oe. The studies are crucial for the development of a new generation of magnetoresistance sensors, such as tunneling magnetoresistance sensors for high-field magnetic sensing, and the future design of a magnetofluidic platform for highly-sensitive detection and analysis of ferrofluid emulsion droplets.
